# Chimeric Porcine Parvovirus VP2 Virus-like Particles with Epitopes of South African Serotype 2 Foot-and-Mouth Disease Virus Elicits Specific Humoral and Cellular Responses in Mice

**DOI:** 10.3390/v16040621

**Published:** 2024-04-17

**Authors:** Qian Li, Xusheng Ma, Yaner Shen, Junfei Dai, Xiaofeng Nian, Xiaofen Shang, Jiao Chen, Ashenafi Kiros Wubshet, Jie Zhang, Haixue Zheng

**Affiliations:** 1 State Key Laboratory for Animal Disease Control and Prevention, Lanzhou Veterinary Research Institute, College of Veterinary Medicine, Lanzhou University, Chinese Academy of Agricultural Sciences, Lanzhou 730046, China; qianli985@163.com (Q.L.); maxushengtt@163.com (X.M.); aixinjueluofei@hotmail.com (J.D.); 18298377925@139.com (X.S.); m18166575233@163.com (J.C.); nafikw@gmail.com (A.K.W.); 2 College of Veterinary Medicine, Northeast Agricultural University, Harbin 150030, China; 3 Gansu Province Research Center for Basic Disciplines of Pathogen Biology, Lanzhou 730046, China; 4 China Agricultural Vet Biologyand Technology Co., Ltd., Lanzhou 730046, China; shenyaner183@163.com; 5 China-Malaysia National Joint Laboratory, Biomedical Research Center, Life Science and Engineering College, Northwest Minzu University, Lanzhou 730030, China; cuicuinian@163.com; 6 Hebei Key Laboratory of Preventive Veterinary Medicine, College of Animal Science and Technology, Hebei Normal University of Science and Technology, Qinhuangdao 066004, China

**Keywords:** chimeric VLP, Epitope, FMDV, PPV, SAT2 serotype

## Abstract

Southern Africa Territories 2 (SAT2) foot-and-mouth disease (FMD) has crossed long-standing regional boundaries in recent years and entered the Middle East. However, the existing vaccines offer poor cross-protection against the circulating strains in the field. Therefore, there is an urgent need for an alternative design approach for vaccines in anticipation of a pandemic of SAT2 Foot-and-mouth disease virus (FMDV). The porcine parvovirus (PPV) VP2 protein can embed exogenous epitopes into the four loops on its surface, assemble into virus-like particles (VLPs), and induce antibodies and cytokines to PPV and the exogenous epitope. In this study, chimeric porcine parvovirus VP2 VLPs (chimeric PPV-SAT2-VLPs) expressing the T-and/or B-cell epitopes of the structural protein VP1 of FMDV SAT2 were produced using the recombinant pFastBac™ Dual vector of baculoviruses in Sf9 and HF cells We used the Bac-to-Bac system to construct the recombinant baculoviruses. The VP2-VLP--SAT2 chimeras displayed chimeric T-cell epitope (amino acids 21–40 of VP1) and/or the B-cell epitope (amino acids 135–174) of SAT FMDV VP1 by substitution of the corresponding regions at the N terminus (amino acids 2–23) and/or loop 2 and/or loop 4 of the PPV VP2 protein, respectively. In mice, the chimeric PPV-SAT2-VLPs induced specific antibodies against PPV and the VP1 protein of SAT2 FMDV. The VP2-VLP-SAT2 chimeras induced specific antibodies to PPV and the VP1 protein specific epitopes of FMDV SAT2. In this study, as a proof-of-concept, successfully generated chimeric PPV-VP2 VLPs expressing epitopes of the structural protein VP1 of FMDV SAT2 that has a potential to prevent FMDV SAT2 and PPV infection in pigs.

## 1. Introduction

Foot-and-mouth disease (FMD) is one of the most economically harmful diseases in the world’s livestock-rich countries, mainly because there are multiple serotypes and topotype of Foot-and-mouth disease virus (FMDV). FMDV is a member of the genus *Aphthovirus* in the family *Picornaviridae*, and causes acute diseases of cattle, sheep, and other cloven-hoofed animals [1]. The virus is classified into seven serotypes (O, A, C, Asia 1, Southern African Territories 1 (SAT1), Southern African Territories 2 (SAT2), and Southern African Territories3 (SAT3)). SAT1, SAT2, and SAT3 are mainly prevalent in Africa [2]. Among SAT1-3 of FMDV, the incidence of SAT2 is the highest. SAT2 FMDV has also shown the fastest transmission rate, multiple transmission channels, and worldwide spread [3]. The distribution of FMDV samples collected in Africa in 2000–2010 indicated that 41% of outbreaks were caused by SAT2. Many reports have confirmed that SAT2 FMDV has crossed geographic boundaries to invade the Middle East. For instance, in 2000, outbreaks of SAT2 foot-and-mouth disease (FMD) were reported in Kuwait and Saudi Arabia, which greatly threatened the global animal husbandry industry. The reports of the World Organization for Animal Health/ Food and Agriculture Organization of the United Nations (WOAH/FAO) Foot-and-Mouth Disease Reference Laboratory Network noted the presence of FMD in North Yemen (1990), Saudi Arabia (2000), KwaZulu-Natal, South Africa (2001), Libya (2003), Bahrain, and Egypt (2012), and more recently in Mauritania, Botswana, Zimbabwe, Nigeria, Kenya, and Ethiopia (2015–2017) [4]. SAT2 FMDV has spread to Arabian Peninsula and the Palestinian Autonomous Territories [5]. There have been outbreaks in the Middle East and Africa in the last two years, including SAT 2 outbreaks in provinces of KwaZulu-Natal and Free State [6]; the most recent outbreak occurred in Zarqa of Jordan or in Iraq in 2023 [7]. Thus, has crossed geographical boundaries, infiltrated the Middle East, and potentially causing a global outbreak [3]. In Asia, due to the high breeding density of pigs and cattle, therefore, the development of a SAT2 FMDV vaccine is urgently required to prevent the spread of SAT2 FMDV.

Determining the antigenic epitopes of viral proteins is an important part of vaccine development. Multiple antigenic epitopes residing on the structural proteins of SAT2 FMDV have been identified [8]. Among these proteins, FMDV VP1 is the most widely studied of these proteins, and sites in the amino acid region 140–150 and 166–170 of VP1 from different strains contain insertions and/or deletions [9]. Notably, Wilhelm et al., identified an FMDV-specific T-cell epitope at amino acids 66–80 of VP1, crucial in cattle immunity against FMDV [10]. Another study has demonstrated that residues 2, 25, 40, 48, and 64 of a monoclonal antibody (monoclonal antibody, mAb) reacted with linear epitope on the FMDV VP1 G-H loop, and that an alteration in the amino acid at positions 149 (valine to glutamine) affected a conformational epitope [8]. The residue 76 (isoleucine or valine) and residue 80 (phenylalanine) may be two specific neutralization epitopes of SAT2 FMDV VP1 [11]. A chicken single-chain antibody fragment was used to identify potential antigenic sites on SAT2 FMDV [12]. Amino acid residue 159 of VP1 protein is not only exposed on the protein surface but is also located within a known immunogenic region [12]. Emad et al. showed that mutated sites in VP1 of the SAT2 FMDV strain prevalent in Egypt in 2013–2014 were mainly concentrated in the regions defined by amino acids 131–149, 156–166, and 206–212 [13].

There is a T-cell epitope at amino acids 21–40 of of FMDV [8]. Amino acids 141–160 (G-H loop) and 200–213 (C-terminal) of VP1 are important B-cell epitopes, and also the sites at which the antigenic residues are most likely vary [8]. With reference to the T- and B- cell epitope of type O FMDV VP1 protein and the already identified epitopes of SAT2 FMDV antigens, we selected two epitopes (T-cell epitope: amino acids 21–40; B-cell epitope: amino acids 135–174) of SAT2 FMDV VP1 for this study.

Porcine parvovirus (PPV) is an extremely stable virus that causes reproductive disorders in swine, which can persist for decades [14,15,16,17]. A sequence alignment analysis showed that the differences between PPV strains all occur in the coding region of the VP2 gene [18]. The PPV VP2 gene determines its hemagglutinin activity, host range, and species evolution [19,20,21]. PPV VP2 protein not only assembled into VLPs, but also induced a specific immune response and neutralization reaction [22]. Therefore, PPV-VP2 VLPs potentially function as vaccines against reproductive failure in pregnant swine. The Escherichia coli-expressed VP2-VLPs induced neutralizing antibody titers similar to those of commercially available inactivated PPV vaccines [23]. Martínez et.al showed that VLPs containing VP2 expressed in insect cells exerted a good protective effect in animal experiments [24]. Another report showed that the absence of loop 2 allowed normal assembly of VLPs, suggesting that loop 2 of VP2 can accommodate foreign proteins, and still permit the assembly of chimeric VLPs [25]. PPV-VLPs carrying Porcine circovirus 2 (PCV2) epitopes (amino acids 165–200 of nucleoprotein), expressed from an adenovirus vector, offers a new strategy for the prevention of diseases associated with PCV2 and PPV co-infection [26]. When the N-terminal of the glycine-rich region of VP2 of PPV was deleted, the VLPs assembled correctly and their immunogenicity was unaffected [27]. Pan successfully prepared chimeric VLPs by inserting the B-cell epitopes of O-type FMDV into loop 2 of VP2 of PPV, and sows vaccinated with an inactivated PPV vaccine or with the VP2-VLPs showed the induction of both humoral and cellular immune responses. Moreover, the cellular immune response of the VP2-VLP-immunized sows was superior to that of sows vaccinated with the inactivated vaccine [28].

In this study, the exogenous T- and/or B-epitopes of VP1 of SAT2 FMDV were embedded in loops of the PPV VP2 protein. Single or tandem repeats of the T-cell epitope (VP1: amino acids 21–40) and/or B-cell epitope (VP1: amino acids 135–174) were successfully constructed to replace the N-terminal and/or loop regions of the PPV VP2 protein. Four chimeric VLPs (chimeric PPV-SAT2-VLPs) were expressed from recombinant baculoviruses in Sf9 or HF cells. In mouse immunization experiments, the chimeric PPV-SAT2-VLPs induced specific antibodies directed against PPV and SAT2 FMDV VP1. The response of mouse splenic lymphocytes to stimulation was significantly enhanced after the mice were immunized with the chimeric PPV-SAT2-VLPs. The number of CD4^+^ and CD8^+^ T lymphocytes in peripheral blood of mice increased significantly, and the secretion levels of interleukin 2 (IL-2), IL-4 and interferon γ (IFN-γ) in serum were also up-regulated compared with the negative control group. In conclusion, we successfully fabricated chimeric VLPs presenting SAT2 FMDV B and T cell epitopes using the Bac-to-Bac system. Animal experiments have shown that at different locations of PPV particles, these molecules are able to stimulate different branches of the immune system to respond. This study created conditions for PPV-VLPs to be used as a carrier molecule to deliver antigens, and in addition, the experiment developed a new strategy to prepare SAT2 FMD virus vaccine, which is expected to prevent SAT2 FMD virus infection.

## 2. Materials and Methods

### 2.1. Reagents, Cells, and Animals

All chemical reagents and biological materials of the highest analytical grade were obtained from international and local commercial companies. The DNA marker, DNA restriction enzymes, *E. coli* DH10Bac cells, *Escherichia coli* DH5α cells, and T4 DNA ligase were purchased from TaKaRa Bio Inc. (Dalian, China). All primers were synthesized by Tsingke (Xi’an, China). All antibodies were purchased as follows: horseradish peroxidase (HRP)-labeled anti-pig immunoglobulin (IgG) and anti-rabbit immunoglobulin (IgG) (Abcam, Cambridge, UK), HRP-labeled anti-rabbit IgG, Alexa Fluor^®^-488-conjugated anti-pig IgG (H+L), and Alexa-Fluor^®^-594-conjugated anti-rabbit IgG (H+L) (Sigma-Aldrich, St. Louis, MO, USA); while antibodies directed against SAT2 FMDV B- (named αVYT) and T-cell epitopes (named αNVQ) were prepared by GenScript (Piscataway, NJ, USA). Several other reagents and chemicals are mentioned in this article with their original sources. PPV strain AV30 was obtained from the Chinese Institute of Veterinary Drug Control (BeiJing, China). Sf9 and HF cells are maintained in our laboratory. Forty-eight female BALB/c mice (6–8 weeks old) were provided by the Lanzhou Veterinary Research Institute, Chinese Academy of Agricultural Sciences (Lanzhou, China) under animal qualification certificate SCXK (G)-2015-0001. The inactivated PPV vaccine (WH-1 strain) was purchased from China Animal Husbandry Industry Co., Ltd. (Beijing, China).

### 2.2. Construction of Chimeric Recombinant Donor Plasmid

**Figure 1:**
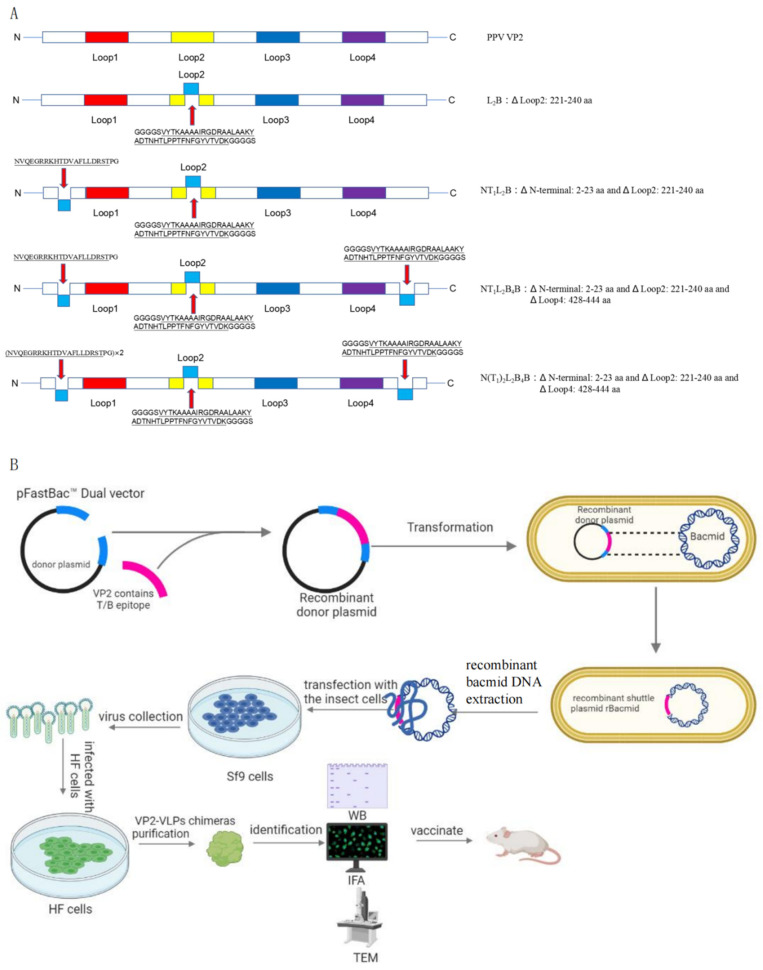
The strategy of inserting SAT2 FMDV VP1 T/B epitopes into PPV VP2 protein and the flow chart of this study. (**A**) the SAT2 FMDV VP1 T/B epitopes insertion positions in the PPV VP2 protein. (**B**) This diagram depicts the generation, expression and identification of target genes of recombinant baculoviruses using the Bac-to-Bac expression system and the immune effect of the immunization of the chimeric proteins in mice. Briefly, VP2 or VP2 with SAT2 FMDV T/B epitopes was inserted into pFastBac^TM^ Dual vector. After DH10Bac transformation and blue and white plaques screening, the recombinant positive shuttle plasmid was transfected into SF9 cells. After observation of the cytopathic changes, recombinant baculovirus were collected and infected with HF cells. The expression and assembly of purified recombination proteins were identified by WB, IFA and TEM, then vaccinated with mice.

The bioinformatics software Primer 5.0 (Canada Primer Premier, San Francisco, CA, USA) was used to design the primers used to amplify the target sequences. The primer sequences are shown in Table 1. The plasmids containing the optimized genes were extracted using the kit (Promega Corporation, Madison, WI, USA). The genes were amplified and cloned into the pFastBac™ Dual vector (Thermo Fisher Scientific Inc., Waltham, MA, USA). The correctly sequenced recombinant plasmids were designated pFastVP2, pFastL_2_B, pFastNT_1_L_2_B, pFastNT_1_L_2_B_4_B, and pFastN(T_1_)_2_L_2_B_4_B. We used a bioinformatic technology (Protein Homology/analogY Recognition Engine V 2.0) to simulate the protein surface structure of the SAT2-specific B-cell epitope of FMDV (isolate PAT/1/2012) VP1 amino acids 135–174: VYTKAAAAIRGDRAALAAKYADTNHTLPPTFNFGYVTVD) and the T-cell epitope (PAT/1/2012 VP1 amino acids 21–40: NVQEGRRKHTDVAFLLDRST), which were then embedded within the PPV AV30 VP2 protein. The following is the construction strategy of these plasmids: L_2_B (B epitope replaced of loop 2 (221–240aa) of PPV VP2), NT_1_L_2_B (T epitope replaced N-terminal (2–23aa) of PPV VP2 and B epitope replaced loop 2 (221–240aa) of PPV VP2), NT_1_L_2_B_4_B (T epitope replaced N-terminal (2–23aa) of PPV VP2 and B epitope replaced loop 2 (221–240aa) of PPV VP2, and B epitope replaced loop 4 (428–444aa) of PPV VP2), and N(T_1_)_2_L_2_B_4_B (two T epitopes replaced the region of 2–23aa of N-terminal of PPV VP2 and B epitope replaced loop 2 (221–240aa) of PPV VP2, and B epitope replaced loop 4 (428–444aa) of PPV VP2) (Figure 1A).

### 2.3. Construction and Identification of Recombinant Shuttle Plasmid

Competent DH10Bac cells transformed with the donor plasmid pFastVP2, pFastL_2_B, pFastNT_1_L_2_B, pFastNT_1_L_2_B_4_B, or pFastN(T_1_)_2_L_2_B_4_B were screened with blue–white selection, and their DNA extracted. The M13F/R primers (Table 1) were used to PCR amplify the recombinant genes in the positive clones, which were designated as rBacmidVP2, rBacmidL_2_B, rBacmidNT_1_L_2_B, rBacmidNT_1_L_2_B_4_B, and rBacmidN(T_1_)_2_L_2_B_4_B, respectively.

### 2.4. Acquisition of Recombinant Baculoviruses

According to the instructions of the manufacturer, Sf9 cells were transfected with the recombinant shuttle plasmids of rBacmidVP2, rBacmidL_2_B, rBacmidNT_1_L_2_B, rBacmidNT_1_L_2_B_4_B, and rBacmidN(T_1_)_2_L_2_B_4_B with Cellfectin^®^ II Reagent (Thermo Fisher Scientific Inc., USA), respectively. A liposome transfection reagent, after cytopathogenic changes were observed, the supernatants were collected and the recombinant viruses isolated and designated as rBacVP2, rBacL_2_B, rBacNT_1_L_2_B, rBacNT_1_L_2_B_4_B, and rBacN(T_1_)_2_L_2_B_4_B, respectively.

### 2.5. Western Blotting and Immunofluorescence Assay (IFA)

HF cells were inoculated with rBacVP2, rBacL_2_B, rBacNT_1_L_2_B, rBacNT_1_L_2_B_4_B, or rBacN(T_1_)_2_L_2_B_4_B. At 72 h after infection, the cells were collected by centrifugation and analyzed with western blotting. Phenylmethylsulfonyl fluoride (PMSF, a protease inhibitor) was added at a concentration of 1% (*v*/*v*) and the cells treated with ultrasound. The supernatants were collected with centrifugation at 16,000× *g* for 10 min and analyzed with western blotting. The blots were incubated with PPV-positive serum or polyclonal rabbit antibodies directed against VYT (αVYT) and NVQ (αNVQ) primary antibodies. The blots were then incubated with the secondary antibodies (HRP-labeled anti-pig IgG antibody or HRP-labeled anti-rabbit IgG antibody). Protein samples from normal HF cell were treated in parallel as the negative controls. At 12 h after infection, the cells were fixed for indirect IFA. The samples were incubated with primary antibody (PPV-positive serum [laboratory preparation] or rabbit polyclonal antibodies directed against the SAT2 FMDV B- or T-cell epitope) and then with secondary antibody (HRP-labeled anti-pig IgG or HRP-labeled anti-rabbit IgG antibody). The secondary antibodies used for IFA were Alexa-Fluor^®^-488-conjugated anti-pig IgG (H+L) and Alexa-Fluor^®^-594-conjugated anti-rabbit IgG (H+L) antibodies. Normal HF cells were treated in parallel as the negative control and αIgG used as a negative antibody control.

### 2.6. VLP Purification and Observation with Transmission Electron Microscopy (TEM)

After infecting HF cells with rBacVP2, rBacL_2_B, rBacNT_1_L_2_B, rBacNT_1_L_2_B_4_B, and rBacN(T_1_)_2_L_2_B_4_B, the cells were collected and lysed, and the supernatant was taken for VLP purification. VLP purification was performed with a linear sucrose density gradient of 15–45%. When the number of HF cells was about 3 × 10^6^ cells/mL, the survival rate of rBacVP2, rBacL_2_B, rBacNT_1_L_2_B, rBacNT_1_L_2_B_4_B and rBacN(T_1_)_2_L_2_B_4_B was reduced to 80%, and the diameter of the cells was about 17.2 μm, the culture was stopped. After the collected cells were broken by ultrasound, the supernatant was collected by centrifugation. A solution with a gradient of 15% to 45% sucrose density was prepared, and the supernatant was slowly added to the upper layer. After centrifugation (30,000 rpm/min) for 4 h, samples were carefully collected with a syringe (1 mL/time) and placed on ice. Choose a sample with a high protein concentration to prepare the electron microscope sample. Drop protein into the center of the copper mesh and leave at room temperature for 15 min, carefully blotting the liquid from the mesh. Stain with 2% sodium phosphotungstic acid solution for 3 min and allow the screen to dry at room temperature. The samples (recombinant VLPs and purified PPV AV30 virions) were placed on a sample rod in a transmission electron microscope (TEM) (Hitachi Co., Tokyo, Japan) and placed in an electron microscope. After focusing, the samples were observed under low power transmission electron microscopy. The field of interest was moved to the screen and then the current of the transmission electron microscope intermediate lens was adjusted to determine the magnification. Focus the image on the fluorescent screen clearly, then take a picture and save it.

### 2.7. Immunization of Mouse Groups

Forty-eight specific-pathogen-free female BALB/c mice aged 6–8 weeks were randomly divided into eight groups. Three rounds of immunizations were given, 14 days apart. Tail vein blood was collected at 7 days post-immunization. After that, the blood was collected every 7 days until the 42nd day, and the serum was separated and stored at −70 °C for further analysis.

### 2.8. Enzyme-Linked Immunosorbent Assays (ELISAs)

The titers of the antibodies directed against the VLP-injected mice were determined with indirect ELISAs. ELISA plates were coated with purified PPV AV30 virions or purified SAT2 FMDV VP1 protein as antigens. The titers of antibody reactivity were recorded as the optical density at a wavelength of 450 nm (OD_450_).

Serum samples were used for the analysis of cytokines. The levels of IL-2, IL-4, and IFN-γ were measured separately with commercial QuantiCyto^®^ Mouse IL-2/IL-4/IFN-γ ELISA kit- (NeoBioscience, Shenzhen, China), according to the manufacturer’s protocols. The plates were read on a Thermo Scientific™ Multiskan™ Skyhigh Microplate Spectrophotometer (Thermo Fisher Scientific, Waltham, MA, USA) at a wavelength of 450 nm.

### 2.9. Lymphocyte Proliferation Assay

Lymphocytes were isolated from specific-pathogen-free mouse spleens using the Spleen Lymphocyte Isolation Kit (Solebol Reagent Co., Ltd., Beijing, China). The cells were seeded in 96-well plates at a concentration of 3 × 10^5^ cells per well and stimulated with 10 μg/mL stimulatory antigen (purified recombinant proteins VP2-VLP, L_2_B, NT_1_L_2_B, NT_1_L_2_B_4_B, or N(T_1_)_2_L_2_B_4_B, or PPV AV30 viral particles). After incubation for 72 h, CCK-8 solution (APExBIO, Houston, TX, USA) was added and the cells were incubated for 4 h. The OD_490_ values were determined with a microplate reader. Concanavalin A (ConA, 5 μg/mL) and RPMI-1640 (Thermo Fisher Scientific Inc., USA) were used as the positive and negative controls, respectively, with six replicates in each group.

### 2.10. Peripheral Blood Flow Test

Fluorescein isothiocyanate (FITC)-conjugated anti-CD4, phycoerythrin (PE)-conjugated anti-CD8, or allophycocyanin (AP)-conjugated anti-CD3 antibody was added to the collected anticoagulated blood (after lysis of red blood cells). A blank control sample was used to adjust the voltage. Then the samples were subjected to test by CytoFLEX LX (Beckman Coulter, Brea, CA, USA) flow cytometer.

### 2.11. Statistical Analysis

GraphPad Prism version 8.0 software (Graph Pad Software Inc., San Diego, CA, USA) was used for all data analyses. *p* ≤ 0.05 on a *t*-test was deemed to indicate a statistically significant difference between variables.

## 3. Results

### 3.1. Designation of Chimeric PPV-SAT2-VLPs with SAT FMDV VP1 Epitopes

In this study, We focused on the invasive South African serotype of FMDV because it has recently been recognized as a serious threat to livestock and pig farming worldwide. The strain we selected is a newly emergent SAT2 serotype, which caused a devastating outbreak of FMD in Egypt in 2012. With reference to the epitopes of type O FMDV VP1 protein and the epitopes of SAT2 FMDV antigens already identified, we selected two epitopes (B-cell epitope 135–174: VYTKAAAAIRGDRAALAAKYADTNHTLPPTFNFGYVTVD; T-cell epitope 21–40: NVQEGRRKHTDVAFLLDRST) for this study. Three suitable embedding sites were finally selected: the N-terminal region, loop 2, and loop 4 of PPV VP2. (Figure 1A). The corresponding genes were synthesized and sequenced by GenScript. The experimental process of the study refers to Figure 1B. The figure depicts the use of the Bac-to-Bac expression system to generate chimeric proteins and immunize mice. Briefly, VP2 or VP2 with the SAT2 FMDV T/B epitope was inserted into the pFastBac^TM^ Dual vector. Recombinant baculoviruses were collected after screening and transfection into SF9 cells to infect HF cells. Expression and assembly of purified recombinant proteins were characterized by WB, IFA, and TEM, followed by inoculation with mice.

### 3.2. Construction of Recombinant Baculoviruses

**Figure 2:**
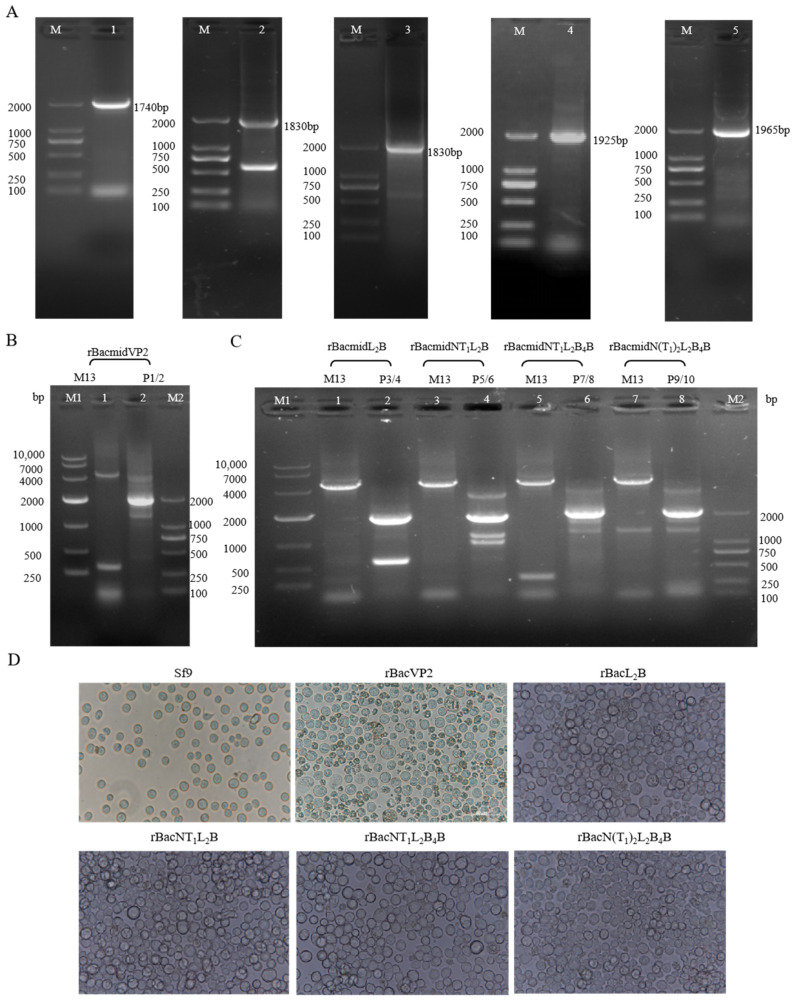
Construction of recombinant baculoviruses. (**A**) PCR amplification of the target gene. lane 1: PCR-amplified VP2 gene; lane 2: PCR-amplified L_2_B gene; lane 3: PCR-amplified NT_1_L_2_B gene; lane 4: PCR-amplified NT_1_L_2_B_4_B gene; lane 5: PCR-amplified N(T_1_)_2_L_2_B_4_B gene. M represents DNA DL 2000bp Marker. (**B**,**C**) Identification of recombinant shuttle plasmids with PCR. (**B**) PCR amplification of rBacmidVP2 gene; Lane 1: M13 primer amplified PCR product; lane 2: P1/2 primer amplified PCR product; (**C**) PCR amplificat the chimeric VP2 genes of rBacmidL_2_B, rBacmidNT_1_L_2_B, rBacmidNT_1_L_2_B_4_B, and rBacmidN(T_1_)_2_L_2_B_4_B. Lanes 1, 3, 5, 7: M13 primer amplified PCR products; lanes 2, 4, 6, 8: P3/4, P5/6, P7/8, P9/10 primer of each gene amplified PCR products. M1 and M2 represent DNA DL 10,000 bp or 2000 bp Marker. (**D**) Morphological changes in Sf9 cells infected with recombinant baculoviruses (400×). Sf9 cells were infected with rBacVP2, rBacL_2_B, rBacNT_1_L_2_B, rBacNT_1_L_2_B_4_B, or rBacN(T_1_)_2_L_2_B_4_B. The morphology of Sf9 cells under a microscope; the diameters of the Sf9 cells increased relative to that of the control.

The target genes were amplified and inserted separately into the insect baculovirus expression vector pFastBac^TM^ Dual. PCR amplification (Figure 2A) showed that the B and T cell epitopes chimeric recombinant genes were correctly inserted into the expression vector. DNA sequencing confirmed the presence of the specific target genes in the pFastBac™ Dual vector. The shuttle plasmid was amplified and identified with the M13 primers. The results showed that the positive competent cells contained the target plasmids (Figure 2B,C). The sequences of the PCR products were consistent with the result shown in Figure 2B,C. Sf9 cells were transfected with the recombinant donor plasmids and cytopathic changes were clearly observed (bloated and rounded cells with inconspicuous nuclei), as shown in Figure 2D.

### 3.3. Identification of Recombinant Protein Expression

**Figure 3:**
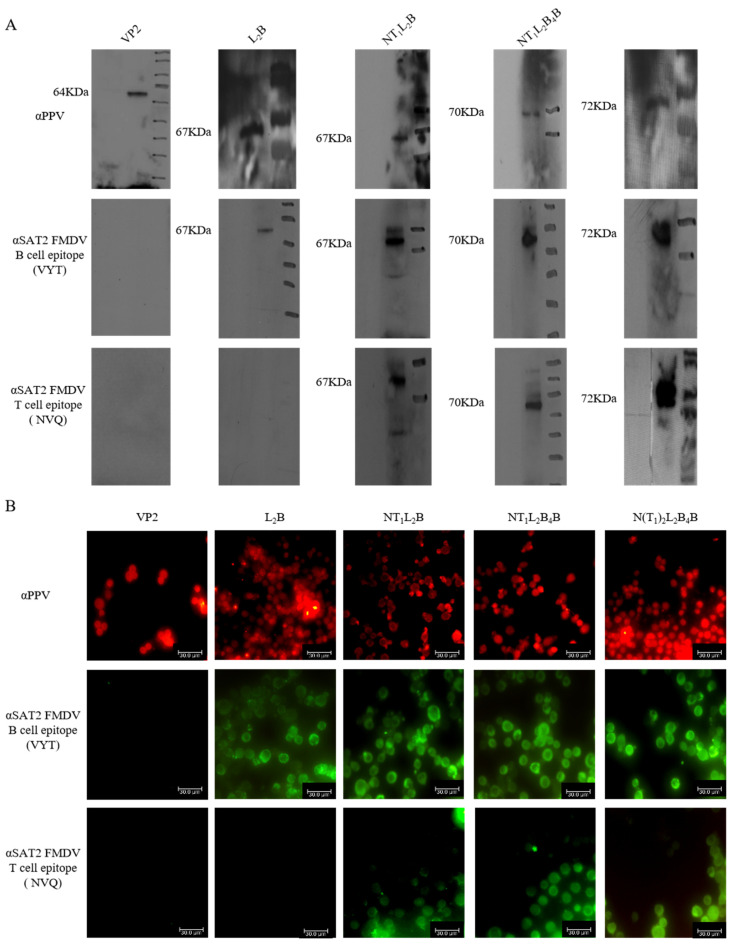
Recombinant proteins were identified by western blotting and an immunofluorescence assay (IFA). (**A**) The expression of the recombinant proteins in HF cells was detected with western blotting. Porcine parvovirus (PPV) VP2 protein and the SAT2 FMDV B- and T-cell epitopes embedded VP2 proteins were detected. (**B**) VP2 protein or recombination VP2 proteins in HF cells were incubated with specific antibodies and observed with IFA (400×).

HF cells (3 × 10^6^/mL, viability > 98%) were inoculated separately with the P3 generations of the recombinant baculoviruses rBacVP2, rBacL_2_B, rBacNT_1_L_2_B, rBacNT_1_L_2_B_4_B, and rBacN(T_1_)_2_L_2_B_4_B. The recombinant proteins were tested with western blotting and IFA, and identified with PPV-positive serum and anti-SAT2 FMDV B- and T-cell epitope antibodies (αVYT and αNVQ, respectively). The VP2 and chimeric VP2 proteins were observed in the infected HF cells as specific bands of 64, 67, 70, and 72 kDa (Figure 3A), suggesting that the VP2 protein and the chimeric proteins containing its B- and/or T-cell epitopes were successfully expressed in HF cells. The recombinant proteins were also identified with IFA. As shown in Figure 3B, compared to the negative anti-IgG (αIgG) control, all the infected HF cells expressed VP2 protein (red fluorescence) after antibody of anti-PPV (αPPV) incubation. Furthermore, using antibodies of anti-SAT2 FMDV B cells epitope (NYT) or anti-SAT2 FMDV T cells epitope (NVQ), all the SAT2 FMDV B-cell epitope chimeras were observed in the infected HF cells (green fluorescence), except rBacVP2. SAT2 FMDV T-cell epitope chimeras NT_1_L_2_B, NT_1_L_2_B_4_B, and N(T_1_)_2_L_2_B_4_B were also observed in the infected HF cells (green fluorescence), but rBacL_2_B was not (Figure 3B). These results suggest that most chimeric SAT2 FMDV B- and T-cell epitopes were successfully expressed in HF cells.

### 3.4. Observation of Chimeric PPV-SAT2-VLPs

**Figure 4:**
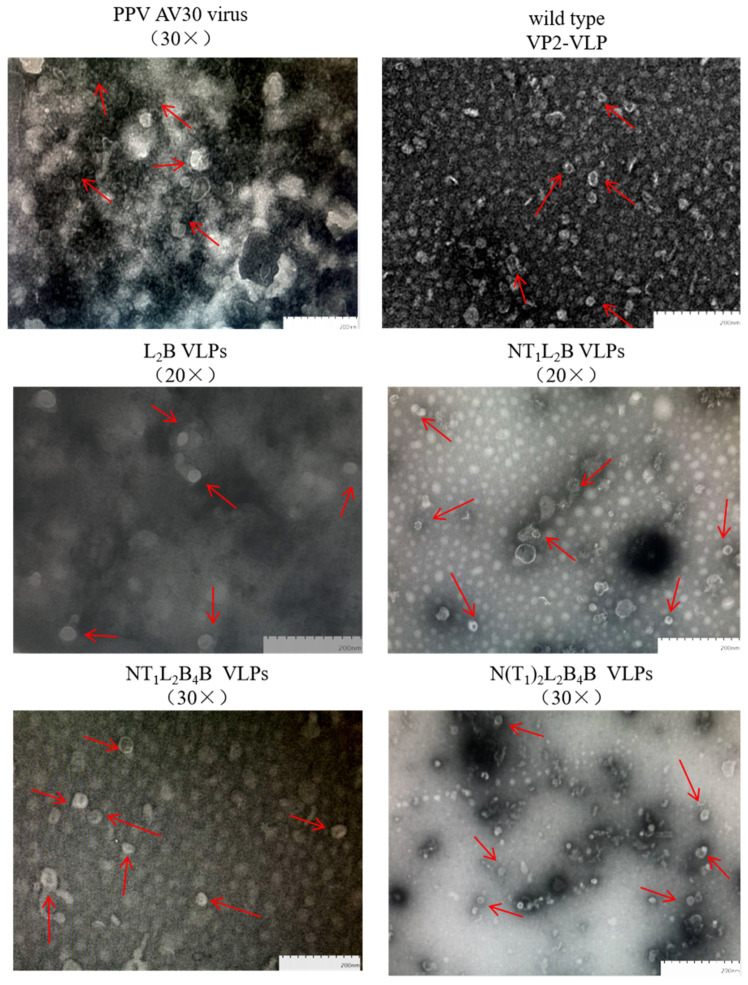
Transmission electron microscopy (TEM) of PPV AV30 and chimeric PPV-SAT2-virus-like particles (VLPs).

TEM images of the purified recombinant VP2-VLP-SAT2 chimeras showed about 30 nm in diameter, and the chimeric VLPs were similar in size and shape to the PPV AV30 virions (control) and wildtype VP2-VLP (Figure 4). These results suggest that the PPV VP2-VLP chimeras containing the SAT2 FMDV B- and/or T-cell epitopes completed the assembly process, and that the insertion of the epitopes did not affect the assembly of PPV VP2 virus-like particles in HF cells.

### 3.5. Fusion Proteins Induced Highly Specific Antibodies in Mice

**Figure 5:**
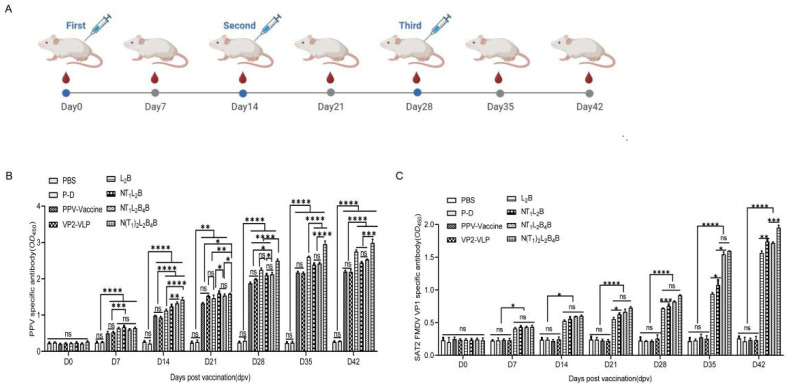
A schematic diagram of mice experiment (**A**) and the specific antibody assessment (**B**,**C**). The results shown are the means and standard deviations of triplicate samples (ns (no significance), * *p* < 0.05, ** *p* < 0.01, *** *p* < 0.001 or **** *p* < 0.0001). ELISA 96-well plates were coated with PPV AV30 virus particles or SAT2 FMDV VP1 protein. Serum was collected at different time points when the mice were inoculated with PBS, P-D, inactivated PPV vaccine, VP2-VLP, L_2_B, NT_1_L_2_B, NT_1_L_2_B_4_B, or N(T_1_)_2_L_2_B_4_B. ELISA was used to detect PPV (**B**), or SAT2 FMDV VP1 specific antibody (**C**).

ELISAs were used to detect the specific IgG responses induced after the injection of the recombination VP2-VLP-SAT2 chimeras (L_2_B, NT_1_L_2_B, NT_1_L_2_B_4_B, or N(T_1_)_2_L_2_B_4_B) in mouse serum (Figure 5A). As shown in Figure 5B, the serum samples from all the immunized mice produced high levels of PPV-specific antibodies. We noted that the serum levels of specific antibodies in the treatment group continued to increase during the whole immune process. The levels of all of them were significantly higher than those controls in the PBS or P-D (pFastBac^TM^ Dual vector, P-D) group (*p* < 0.05). These data indicate that the fusion proteins induced an effective humoral immune response in mice and that VP2-VLP induced PPV-specific antibodies to the same level as the inactivated vaccine. The PPV-specific antibody levels directed against the chimeric PPV-SAT2-VLPs were significantly higher than those directed against wildtype VP2-VLP, indicating increased after epitope insertion.

As shown in Figure 5C, the levels of specific antibodies against the SAT2 FMDV VP1 protein from all the immunized mice were determined. We noted that the specific serum antibody levels continued to increase in the treatment group throughout the immunization period, and were significantly higher than in the groups treated with PBS, P-D, VP2-VLP, or PPV vaccine (*p* < 0.05). These data indicated that the fusion proteins induced an effective humoral immune response against VP1 in mice. These results also showed that the induction of mode of specific antibody induction by chimeric PPV-SAT2-VLPs was similar to their induction by SAT2 FMDV VP1 (refer to [29]).

### 3.6. Fusion Proteins Increased the Proliferation of Mouse Lymphocytes

**Figure 6:**
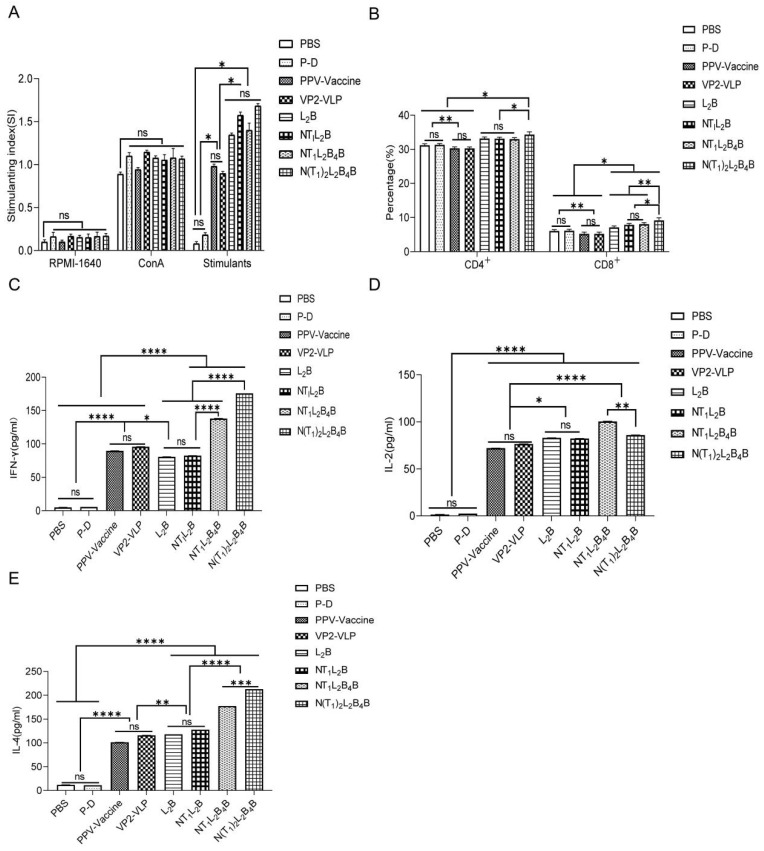
Immunization of recombinant proteins increased CD4^+^ and CD8^+^ T-lymphocyte proliferation and cytokine production in mice. each value (pg/mL) in the bar diagram represents the mean ± SEM (n = 7); ns: no significance, * *p* < 0.05, ** *p* < 0.01, *** *p* < 0.001 and **** *p* < 0.0001 compared with the control group. (**A**) Recombinant proteins increased lymphocyte proliferation. RPMI-1640 was used as the negative control, and ConA (5 μg/mL) was used as the positive control. Lymphocyte count (%) was increased significantly in the recombinant protein stimulation groups (* *p* < 0.05), compared to the control group. The results are presented as the ratio of the OD490 of the stimulated sample to the OD_490_ of the unstimulated sample (stimulation index). (**B**): CD4^+^ and CD8^+^ T cells from peripheral blood were assayed after immunizations with VP2-VLP, PPV vaccine groups, PBS, P-D groups L_2_B, NT_1_L_2_B, NT_1_L_2_B_4_B or N(T_1_)_2_L_2_B_4_B. (C–E): A *t*-test was used to determine the mean variations in the serum levels of cytokines (IFN-γ, IL-4, and IL-2) among the treatment groups and control groups (ns: no significance, * *p* < 0.05, ** *p* < 0.01, *** *p* < 0.001). (**C**) The concentrations of IFN-γ were determined in the eight immunized groups. (**D**) The concentrations of IL-2 in mice were detected after immunizations with recombinant proteins or control. (**E**) The mice were treated with PBS, P-D, PPV-vaccine, VP2-VLP, or recombinant proteins, respectively, then IL-4 level was determined.

The lymphocyte proliferative response to the purified proteins in all groups of immunized mice was analyzed in vitro at 42 days post-injection (dpi). As shown in Figure 6A, significantly higher lymphocyte proliferation was observed in the splenic lymphocytes of mice immunized with the fusion proteins than in the control mice (*p* < 0.01). These results indicate that the fusion proteins effectively stimulated the cellular immune response, which would enhance the humoral response.

### 3.7. CD4^+^ and CD8^+^ T Lymphocytes Were Elevated in the Peripheral Blood of Mice Vaccinated with the VP2-VLP Chimeras

The peripheral blood lymphocytes of mice were isolated at 42 dpi, and the proportions of CD4^+^ and CD8^+^ T lymphocytes among them were analyzed with flow cytometry. The results are shown in Figure 6B. The four chimeric proteins induced both humoral and cellular immune responses in the mice after their intramuscular injection. CD4^+^ and CD8^+^ cells were significantly increased in the VP2-VLP and inactivated PPV vaccine groups compared with those in the PBS and P-D (pFastBac™ Dual vector) groups (*p* < 0.001). The proportion of CD4^+^ T lymphocytes was significantly lower in the L_2_B, NT_1_L_2_B, and NT_1_L_2_B_4_B groups than in the N(T_1_)_2_L_2_B_4_B group (*p* < 0.05). The proportions of CD4^+^ T lymphocytes in the L_2_B, NT_1_L_2_B, and NT_1_L_2_B_4_B groups did not differ significantly (ns). Similarly, the proportion of CD8^+^ T lymphocytes in the N(T_1_)_2_L_2_B_4_B group was also significantly higher than in the NT_1_L_2_B, and NT_1_L_2_B_4_B groups (*p* < 0.001). There was no significant difference in the proportions of CD8^+^ T lymphocytes in the NT_1_L_2_B and NT_1_L_2_B_4_B groups (ns). Moreover, the proportions of CD4^+^ and CD8^+^ T lymphocytes in the L_2_B, NT_1_L_2_B, NT_1_L_2_B_4_B, and N(T_1_)_2_L_2_B_4_B groups were significantly higher than in the VP2-VLP, PPV, PBS, or P-D group (*p* < 0.05). These results indicate that chimeric VP2-VLP not only stimulated the proliferation of CD4^+^ lymphocytes, but also the proliferation of CD8^+^ lymphocytes.

### 3.8. VP2-VLP Chimeras Increased Cytokine Production

To determine the degree of humoral and cellular immunity induced in the immunized mice, the concentrations of Th1 cytokines (IL-2, IFN-γ) and a Th2 cytokine (IL-4) in their sera were determined. The IFN-γ levels were significantly higher in the treatment groups than in the PBS or P-D control group (*p* < 0.0001; Figure 6C). The serum IFN-γ levels were also significantly higher in the VP2-VLP and inactivated PPV vaccine groups than in the PBS and P-D control groups (*p* < 0.05). IFN-γ was significantly higher in the VP2-VLP chimera groups than in the VP2-VLP and PPV inactivated vaccine groups (*p* < 0.05). Furthermore, IFN-γ expression was significantly higher in the N(T_1_)_2_L_2_B_4_B-injected mice than in L_2_B-, NT_1_L_2_B-, or NT_1_L_2_B_4_B-injected mice (*p* < 0.0001).

The IL-4 and IL-2 levels also showed significant variation between the treatment groups and the control groups. As shown in Figure 6D, the serum IL-2 levels were significantly higher in the experimental groups than in the PBS and P-D control groups (*p* < 0.0001), but there was no significant difference between the VP2-VLP group and the inactivated-PPV-vaccinated group. IL-2 secretion was significantly higher in all VP2-VLP-SAT2 chimera groups than in the groups treated with VP2-VLP or the inactivated PPV vaccine. There was no significant difference in the IL-2 levels in the L_2_B group, NT_1_L_2_B group, and NT_1_L_2_B_4_B group (*p* > 0.05). However, it was higher in the N(T_1_)_2_L_2_B_4_B group than in the L_2_B, NT_1_L_2_B, and NT_1_L_2_B_4_B groups (*p* < 0.0001).

As shown in Figure 6E, the IL-4 levels were significantly higher in the experimental groups than in the PBS and P-D control groups (*p* < 0.0001), but did not differ significantly between the L_2_B, NT_1_L_2_B, and NT_1_L_2_B_4_B treatment groups (*p* > 0.05). However, IL-4 expression was significantly higher in all the groups treated with a VP2-VLP chimera than in the VP2-VLP and inactivated PPV treatment groups (*p* < 0.05), and N(T_1_)_2_L_2_B_4_B induced higher IL-4 expression than L_2_B, NT_1_L_2_B, or NT_1_L_2_B_4_B (*p* < 0.001).

## 4. Discussion

The development of vaccines of antigen epitopes ensures that the prevention and control using epidemic diseases are both effective and safe. FMDV VP1 carries essential epitopes inducing protective immune responses in host. Notably, high amino acid sequence variations exist in the major immunogenic sequences of VP1 protein across various FMDV serotypes and subtypes [8]. Specific T-cell epitopes have been identified at amino acids 21–40 and 66–80 of VP1, with the latter playing a pivotal role in proliferation of PBMC from FMDV vaccinated cattle [30]. Additionally, an FMDV-specific T-cell epitope at amino acids 66–80 of VP1 was also identified, which plays a leading role in the immunity of cattle, not pigs, infected with FMDV [10]. Due to the intensive and large number of pig farming in China, we did not choose this T cell epitope. Two major B-cell epitopes located in the region of amino acids 141–160 (G-H ring) and amino acids 200–213 (C-terminus) of VP1, regions prone to antigenic variations [8]. The G-H loop (containing highly conserved Arg-Gly-Asp (RGD) motif) of VP1 on the FMDV capsid surface contributes to the neutralizing antibodies induction in hosts [31].

The antigenicity and immunogenicity of the SAT2 FMDV VP1 proteins remain poorly understood. By referencing B/T cell epitopes of type O FMDV VP1 and known epitopes of SAT2 FMDV antigens [11], we selected SAT2 FMDV structural protein VP1, which contains a B-cell epitope at 141–160aa and a T-cell epitope at 16–40aa for developing an effective vaccine that can induce humoral and cellular immune response in host.

VLPs have been used as vaccines carrying foreign epitopes to combat infectious diseases [32,33]. The PPV VP2 protein plays a major structural role in the capsid of PPV, and its antigenic sites are mainly concentrated in its four loops. After assembly, these VLPs stimulate the production of antibodies and related cytokines in animals. Chimeric PPV-SAT2-VLPs were demonstrated to be used as antigen vehicle carrying type O FMDV epitopes [28]. The titers of the antibodies raised and cytokines induced (IFN-γ, IL-2, and IL-4) in sows immunized with an inactivated PPV vaccine or VP2-VLP prepared in the laboratory indicated that both vaccines induced humoral and cellular immune responses. However, VP2-VLP stimulated the sows to produce a better cellular immune response than the inactivated vaccine [23]. Four loops occur on the surface of PPV VP2 protein, in the regions defined by amino acids 66–101, 212–245, 273–333, and 413–424, and are sites of the epitopes located on the surface of the capsid protein [22]. In a study of these loops, a recombinant protein was embedded in loop 2 of VP2 of canine parvovirus (CPV), in the same family as PPV, and the recombinant virus not only assembled into VLPs, but also induced a specific immune response and neutralization reaction [21]. The multiple epitope-containing gene of O type FMDV VP1 was designed by substituting PPV VP2 loops to form VLPs capable of inducing immune responses in BALB/c mice [28]. However, Pan et al., also found that PPV VP2 Loop4 or loop2-loop4 deletion resulted in a lower number of particles and the morphology of the particles was not well preserved [25]. The N-terminal deletion mutants of VP2 (40 amino acids) of CPV was fused to the enhanced green fluorescent protein (EGFP), which were able to form VLPs [34]. It was reported that the glycine-rich domain of PPV VP2 N-terminal is not essential for VLP assembly [27]. SAT2 FMDV epitopes expressed in PPV VP2-VLP system have not been tested before this study. We added SAT2 FMDV VP1 protein epitopes to replace the N-terminus (2–23aa), loop 2 (221–240aa), and downstream location of loop 4 (428–444aa) regions of PPV VP2. TEM results of these chimeric PPV-SAT2-VLPs indicated that the deletion of the nonessential region of VP2 did not affect on the structure, expression, or assembly of the VP2 protein. We concluded that the insertion of the B-cell epitope into loop 2 of PPV VP2 allowed the VLPs to form, and that the insertion of the T-cell epitope at the N-terminus did not negatively affect on the assembly of the chimeric VLPs. It also suggested that other types of epitopes could be inserted into and expressed from these positions, which may not have negative impact on protein assembly.

In our study, the insect baculovirus expression system was used to express the chimeric PPV VP2 protein carrying epitopes of SAT2 FMDV VP1. The four chimeric PPV VP2 proteins (L_2_B, NT_1_L_2_B, NT_1_L_2_B_4_B, and N(T_1_)_2_L_2_B_4_B) were expressed in Sf9 and HF cells. After purification, the chimeric PPV VP2 proteins were observed to form VLPs under TEMs. Some of the chimeric PPV VP2 formed more irregular VLPs than PPV AV30 virus. This suggests the efficiency of VLP assembly may be different, resulting in some structural irregularities. However, this does not hinder the specific antibody production in mice by the chimeric VP2.

One week after BALB/c mice were immunized with the recombinant protein VP2-VLP, L_2_B, NT_1_L_2_B, NT_1_L_2_B_4_B, or N(T_1_)_2_L_2_B_4_B, specific antibodies directed against PPV and SAT2 FMDV VP1 were produced in their bodies. When they were re-immunized on day 14 after the initial immunization, the antibody levels gradually increased, peaking on day 42 after immunization. The data indicated that the chimeric PPV-SAT2-VLPs as candidate vaccines, which can induce the humoral immune response in host.

Furthermore, chimeric VP2 immunized mice induced significantly higher lymphocyte proliferation, suggesting that these recombinant proteins are able to elicit specific cellular immunity than controls. Determination of the peripheral blood cytokine contents showed that the immunogen N(T_1_)_2_L_2_B_4_B stimulated the production of Th1 cytokines significantly better than the inactivated PPV vaccine. Th1 cells promote the activation and proliferation of macrophages, cytotoxic T lymphocytes, and NK cells by producing cytokines such as IFN-γ and IL-2, thus expanding the cellular immune effect. Thus, it can be tentatively established that the chimeric protein N(T_1_)_2_L_2_B_4_B displays T cell epitopes on the surface during VLP formation and enhances cellular immune responses. The immunogen N(T_1_)_2_L_2_B_4_B also induced a significant increase in the Th2-type cytokine response. Th2 cells promote the proliferation and differentiation of B cells into plasma cells by producing cytokines such as IL-4 and IL-10 and establishing the connection between CD40 of B cells and CD40L of Th2 cells, which induces the B cell to produce antibodies as part of the humoral immune response. It has been suggested that the B-cell epitope inserted into a recombinant chimeric protein played a specific role in mediating the humoral immune response [30]. There was no significant difference between NT_1_L_2_B and NT_1_L_2_B_4_B in cytokine induction, indicating that the B epitope inserted into loop 4 had no significant effect on cytokines secretion. However, compared with the inactivated PPV vaccine and VP2-VLP, the four recombinant chimeric proteins all improved the immune responses with variating degree. The CD4^+^ and CD8^+^ T lymphocytes in the anti-coagulated peripheral mouse blood were significantly elevated relative to those in the negative control group. Taken together, these results suggest that the fusion proteins potentially orchestrated the cellular and humoral immune responses against FMDV and PPV.

Because there has been no outbreak of SAT2 FMDV in China, our experiments on preventive vaccines are related to improving national biosecurity. Thus, we only confirmed the antibody levels and cytokine production induced after immunizations with chimeric VLPs in mice, which was a limitation of the whole study. SAT2 FMDV must be used as the control group for further experimental comparisons, to ensure that a more reliable conclusion can be drawn. In spite of these caveats, our results demonstrated that the PPV VP2 protein carrying VP1 T/B epitopes of SAT2 FMDV expressed in insect baculovirus expression system could form VLPs and induce anti-SAT2 FMDV VP1 antibody response in mice. Thus, the chimeric PPV VP2 could be a candidate antigen for developing novel SAT2 FMDV vaccine.

## 5. Conclusions

In this study, three PPV VP2 sites were successfully embedded with the B- and T-cell epitopes of SAT2 FMDV VP1 protein to assemble in them and the recombinant chimeric PPV VP2 into VLPs. Four purified recombinant chimeric PPV-VP2 proteins were prepared and used immunize mice. These four purified recombinant proteins showed good antigenicity and immunogenicity, and induced effective humoral and cellular immune responses in mice. We believe that these chimeric PPV-SAT2-VLPs could be used as a potential vaccine candidate against SAT2 FMDV infection.

## Figures and Tables

**Table 1:** The primers along with appropriate restriction endonuclease enzyme (underlined in the primer sequence) that amplify the five protein-encoding genes.

Primers	5′-3′ Sequences		Restriction Enzyme
P1/P2	CGGGATCCATGAGTGAAAATGTGGAACAACAC		*BamH* I (Forward primers) *Pst* I (Reverse Primers)
	AACTGCAGCTAGTATAATTTTCTTGG		
P3/P4	CGGGATCCCTGGAACGCTACACTTTCAACCC		
	AACTGCAGTTAGTACAGCTTTCTAGGGATCAGCTGGC		
P5/P6	CGGGATCCATGTCCGAGAACGTGGAACAGCACAACC		
	AACTGCAGTTAGTACAGCTTACGAGGGATCAGCTGGG		
P7/P8	CGGGATCCATGAACGTGCAAGAGGGCCGCCG		
	AACTGCAGTTAGTACAGCTTTCTAGGGATCAGCTGG		
P9/P10	CGGGATCCATGAACGTGCAGGAGGGTCGCCGTAAG		
	AACTGCATTAGTACAGCTTACGAGGGATCAGCTGGC		
P11/P12	CGGGATCCATGAACGTGCAGGAGGGTCGCCGTAAGC		
	AACTGCAGTTAGTACAGCTTACGAGGGATCAGCTGGC		
M13F/R	GTTTTTCCCAGTCACGAC		No restriction site
	CAGGAAACAGCTATGAC		
	Temperature	Time	Cycle
Initial denaturation	95 °C	3 min	1 cycle
1st denaturation	95 °C	30 s	34 cycle
Annealing	55 °C	30 s (5 min)	
Elongation	72 °C	1 min	
Final elongation	72 °C	10 min	1 cycle

## Data Availability

Data are contained within the article.
